# Galactic Cosmic Radiation Leads to Cognitive Impairment and Increased Aβ Plaque Accumulation in a Mouse Model of Alzheimer’s Disease

**DOI:** 10.1371/journal.pone.0053275

**Published:** 2012-12-31

**Authors:** Jonathan D. Cherry, Bin Liu, Jeffrey L. Frost, Cynthia A. Lemere, Jacqueline P. Williams, John A. Olschowka, M. Kerry O’Banion

**Affiliations:** 1 Department of Pathology and Laboratory Medicine, University of Rochester School of Medicine and Dentistry, Rochester, New York, United States of America; 2 Center for Neurologic Diseases, Brigham and Women’s Hospital, Harvard Medical School, Boston, Massachusetts, United States of America; 3 Department of Radiation Oncology, University of Rochester School of Medicine and Dentistry, Rochester, New York, United States of America; 4 Department of Neurobiology & Anatomy, University of Rochester School of Medicine and Dentistry, Rochester, New York, United States of America; University of IIllinois, United States of America

## Abstract

Galactic Cosmic Radiation consisting of high-energy, high-charged (HZE) particles poses a significant threat to future astronauts in deep space. Aside from cancer, concerns have been raised about late degenerative risks, including effects on the brain. In this study we examined the effects of ^56^Fe particle irradiation in an APP/PS1 mouse model of Alzheimer’s disease (AD). We demonstrated 6 months after exposure to 10 and 100 cGy ^56^Fe radiation at 1 GeV/µ, that APP/PS1 mice show decreased cognitive abilities measured by contextual fear conditioning and novel object recognition tests. Furthermore, in male mice we saw acceleration of Aβ plaque pathology using Congo red and 6E10 staining, which was further confirmed by ELISA measures of Aβ isoforms. Increases were not due to higher levels of amyloid precursor protein (APP) or increased cleavage as measured by levels of the β C-terminal fragment of APP. Additionally, we saw no change in microglial activation levels judging by CD68 and Iba-1 immunoreactivities in and around Aβ plaques or insulin degrading enzyme, which has been shown to degrade Aβ. However, immunohistochemical analysis of ICAM-1 showed evidence of endothelial activation after 100 cGy irradiation in male mice, suggesting possible alterations in Aβ trafficking through the blood brain barrier as a possible cause of plaque increase. Overall, our results show for the first time that HZE particle radiation can increase Aβ plaque pathology in an APP/PS1 mouse model of AD.

## Introduction

After more than 50 years of manned space exploration, plans are underway to return to the moon and explore other locations beyond Earth’s protective magnetic field, including asteroids and Mars. This does not come without significant risk. In particular, a major risk factor for human health in deep space is radiation. The galactic environment is dominated by high levels of protons arising from solar flares, and low, but continuous levels of Galactic Cosmic Radiation (GCR) [1]. GCR is made of high-energy, high-charged (HZE) particles that contain a variety of different elements, including ^56^Fe particles [2]. Radiation-induced late degenerative changes represent a potential risk for future astronauts [1], [3]. A significant focus of NASA’s efforts to assess radiation risk has centered on possible late effects in the central nervous system (CNS). For example, similar to more well studied terrestrial radiation such as γ rays [4], ^56^Fe particle radiation has been documented to cause neuroinflammation [5], a clear indicator of CNS damage [6]. Furthermore, even at very low doses, ^56^Fe particle radiation can result in neurogenesis defects and cognitive impairment [5], [7]. Given that there is a high probability of HZE particles hitting CNS neurons during a space mission [2], concerns have been raised regarding the potential effects of space radiation on promoting neurodegenerative disorders, including Alzheimer’s disease (AD), which will afflict as many as 45% of individuals who survive past the age of 85 [8].

AD is characterized by a progressive cognitive decline over several years [9]. This cognitive decline is thought in part, to result from an ongoing chronic neuroinflammatory process [10]. One of the key players in neuroinflammation and one of the two major histopathological hallmarks of the disease is accumulation of amyloid beta (Aβ) into extracellular, dense fibril plaques [11]. Monitoring plaque progression in vivo has been used to gauge disease severity [12] and has recently been approved as a diagnostic tool for human imaging studies [13]. Since the inflammatory environment appears to play a role in driving disease progression [11], any inflammatory changes can alter AD pathology. We, as well as other groups, have shown that exposure of the CNS to various cytokines [14]–[16] or bacterial components [17] can drastically alter plaque pathology depending on the specific stimulus provided. Additionally, there is accumulating evidence that peripheral inflammatory stimuli can also influence Aβ accumulation [18], [19]. This demonstrates that AD pathology is dynamic and sensitive to CNS environmental changes. Inflammation is also associated with neurovascular dysfunction. Furthermore, this dysfunction has been linked to impaired transport of Aβ out of the brain, resulting in increased accumulation and disease progression [20]. Indeed, decreased blood brain barrier (BBB) transport of Aβ has been implicated in mouse and human studies [21]. Interestingly, radiation has also been clearly documented to cause BBB break down and dysfunction [22].

The potential disease-altering effects of GCR prompted us to examine if HZE radiation influences AD pathological progression using an APP/PS1 mouse model that shows age-associated accumulation of Aβ plaques and cognitive dysfunction [23], [24]. We discovered that ^56^Fe particle radiation resulted in cognitive impairment and increased Aβ plaque pathology at cumulative doses similar to those that astronauts might be exposed to on exploratory missions to deep space and Mars [3].

## Materials and Methods

### Ethics Statement

This study was carried out in strict accordance with the recommendations in the Guide for the Care and Use of Laboratory Animals of the National Institutes of Health. Animal protocols were reviewed and approved by the University of Rochester (Protocol Number: 2008–38) and Brookhaven National Laboratory’s (BNL) (Protocol Number: 442) Institutional Animal Care and Use Committees.

### Animals

Twenty-nine male and twenty female APPswe/PSEN1dE9 (APP/PS1) mice (stock no. 004462) on a mixed C3H/HeJ and C57BL/6 background were purchased from The Jackson Laboratory at approximately 3 months of age. Animals were shipped to BNL and allowed to acclimate. Mice were housed five per cage in temperature (23 ± 3°C) and light (12∶12 light:dark) controlled rooms with free access to chow and water. After radiation exposure at 3.5 months of age, animals were shipped back to the University of Rochester until euthanasia. Mice were routinely monitored for health issues and had no observable problems at the time of euthanasia. Male mice were euthanized at 9.5 months of age while female mice were euthanized at 7 months due to concerns raised regarding early death.

### Irradiation

Radiation exposures were performed at NASA’s Space Radiation Laboratory at BNL. Animals were loaded into ventilated 50 mL polystyrene conical tubes and irradiated, 8 at a time, using a foam tube holder positioned at the center of a 20×20 cm beam of iron ions accelerated to 1 GeV/µ at a dose rate ranging from 0.1–1 Gy/min. Male mice received total doses of either 10 cGy or 100 cGy. Female mice received only a 100 cGy dose. Control mice were similarly placed in tubes and sham irradiated.

### Behavioral Testing

Memory was tested using two different paradigms. The first was contextual fear conditioning, which tests the ability of the subject to recognize an environment associated with an adverse stimulus (foot shock). Fear conditioning was set up, performed, and analyzed as previously described [25]. In brief, mice were allowed to explore a novel chamber for 3 minutes, then 15 s of white noise (80 dB) was presented and co-terminated with a 2 s, 0.7 mA foot shock. This noise-shock paring was repeated twice for a total of 3 shocks, using an interval of 30 s between shocks. Twenty-four hours later, mice were placed back into the same chamber and freezing was measured for 5 min. Four hours later, mice were placed in a novel context for 3 min then re-exposed to the white noise (cued tone response) for 3 min and freezing was analyzed. Novel object recognition was preformed with assistance from the University of Rochester Behavioral Science Facility Core. This test was performed and scored as described previously [26]. Our learning trial time was 10 minutes and the testing trial time was 5 minutes with a one hour delay between each trial. The entire first 10 min session was scored while only the first 2 min of the 2^nd^ test session was scored. A recognition index (RI) for time spent with the novel object was calculated based on the proportion of total time spent with the novel object.

### Tissue Collection

Animals were anesthetized and perfused with saline as previously described [16]. The brains were then harvested and the hemispheres were bisected with a razor blade. The right half was fixed in ice cold 4% paraformaldehyde (PFA) while the left half was snap-frozen in isopentane and stored at −80°C until used for ELISA and Western blot analysis. The fixed tissue remained overnight in 4% PFA at 4°C and was then transferred to 30% sucrose until equilibrated.

### Immunohistochemistry (IHC)

Brains were sectioned at 30 µm on a sliding knife microtome with a −25°C freezing stage. Sections were stored in cryoprotectant at −20°C until processing. Antibody staining was visualized using either biotinylated secondary antibodies, avidin-biotin complex (Elite), and a 3,3-diaminobenxadine (DAB) substrate kit (Vector Laboratories) or, immunofluorescent secondary antibodies bound to Alexa fluorophores (Invitrogen) at a dilution of 1∶500. Primary antibodies used were mouse anti-6E10 (Covance, 1∶1000), rabbit anti-GFAP (DAKO 1∶1000), rabbit anti-Iba-1 (Wako, 1∶2000), rabbit anti-CD68 (AbD Serotec, 1∶500), and Armenian hamster anti-ICAM (Thermo Scientific, 1∶1000). Biotinylated secondary antibodies against their proper species (Jackson Laboratory) were used at 1∶1000. For Congo red staining, a kit from Sigma-Aldrich was used.

### Quantification of Amyloid Plaque Load and Glial Activation

Brains sections were viewed with an Axioplan 2i light microscope (Zeiss). For plaque area, a 5x lens was used. Multiple images were taken of a single section to obtain pictures of the whole cortex and hippocampus. Images were merged in Photoshop and subjected to threshold analysis using the max entropy threshold algorithm in NIH ImageJ (V1.46, http://rsbweb.nih.gov/ij/). The percent area occupied by 6E10 or Congo red of the cortex and hippocampus was calculated and analyzed. In addition to the percent area of 6E10, the total number and average size of 6E10 positive plaques was obtained using this threshold algorithm. The percent area occupied by GFAP was calculated for cortex only. Values obtained for male mice were analyzed with a one-way ANOVA followed by Bonferroni post test comparing the different doses. Values for female mice were analyzed with a Student’s t-test.

Microglial activation was analyzed by capturing images at 40x magnification. Images were taken of Congo red stained dense plaques. The images were transferred to NIH ImageJ and the three color channels comprising CD68, Iba-1, and Congo red were separated and viewed individually. A 500 pixel total area circle was placed in the center of each plaque. In total, 6 Congo-red-positive plaques in each of two hippocampal sections were analyzed and averaged together for each mouse. Using the max entropy threshold algorithm we calculated the percent area inside the 500 pixel circle occupied by CD68, Iba1, and Congo red. Prism v5 (Graphpad Software) was used for all statistical analyses. A value of p < 0.05 was considered significant.

### Protein Quantification

Western blot and ELISA protein samples were prepared as previously described [16], [27]. Briefly, half brains were homogenized then sonicated in 1 mL of T-per (Pierce) and protease inhibitor cocktail set I (Calbiochem). 100 µL of homogenized sample was removed and stored at −80°C for Western blot. Remaining samples were centrifuged at 100,000g for 60 minutes. Supernatants (soluble fraction) were removed and the pellet was resuspended in 150 mg/mL Guanidinium HCL pH 8.0 followed by recentrifugation at 100,000g to generate an insoluble fraction. Soluble and insoluble Aβ isoforms were assayed using Invitrogen ELISA kits for Aβ42 and Aβ40 (#KHB3544 and #KHB3841, respectively). T-per soluble fractions were also used for TNFα ELISA (#KMC3011). Protein concentrations for Western blot samples were measured with a Micro BCA protein assay (Thermo Scientific). 15 µg of protein was subjected to SDS-PAGE, transferred to polyvinylidene difluoride, and probed with antibodies specific against the following substrates: Mouse anti-Amyloid Precursor Protein (Covance, 1∶1000), Rabbit anti-β-CTF (Sigma, 1∶1000), Rabbit anti-IDE (Calbiochem 1∶1000), Rabbit anti-LRP1 (Epitomics, 1∶10,000), and α-tubulin (Calbiochem, 1∶5000). Only the male 0 cGy and 100 cGy samples were used for Western blots.

## Results

**Figure 1:**
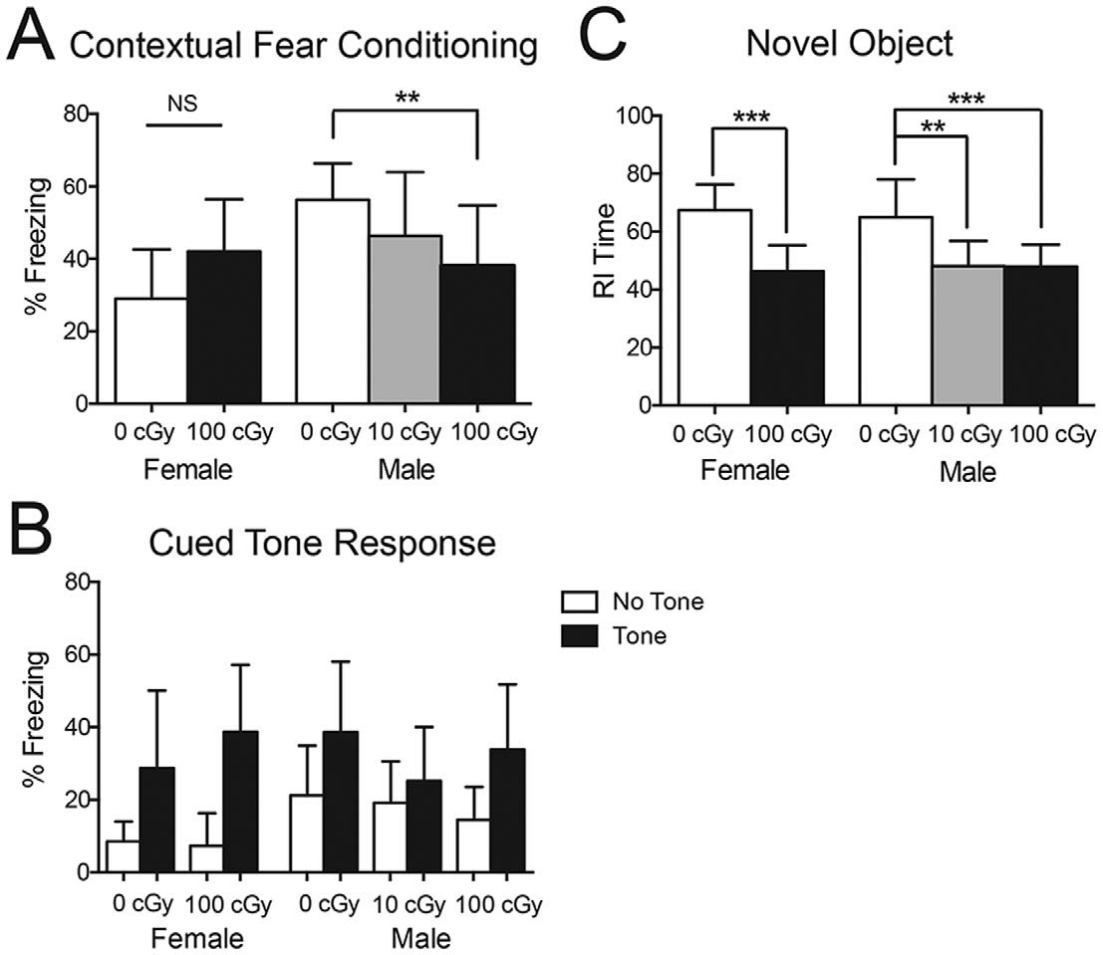
Effect of ^56^Fe particle radiation on memory and cognition using contextual fear conditioning and novel object recognition tests. (*A*) Fear conditioning results quantified as percent time freezing. (*B*) No significant difference was found between any groups in freezing to a novel context or a tone stimulus. (*C*) Novel object recognition test using the recognition index generated for time spent with the novel object. All data is compared within the respective gender. Data was analyzed with Student’s t-test for the females and one-way ANOVA with a Bonferroni post test for the males. Graphs show means ± SD, *n = *8–14 animals per condition at each dose. **P<.01, ***P<.001.

To assess the effect of iron galactic cosmic radiation on memory and cognition two separate tests were employed prior to tissue harvest at 9.5 mo for males and 7 mo for females. To assess hippocampal-dependent memory, the first test used was contextual fear conditioning (Fig. 1*A*). We found an overall significant difference in freezing behavior as measured by one-way ANOVA in the male group [F(2,32) = 5.122, p = .0118] and post-hoc analysis revealed a significant decrease in freezing behavior between the 0 cGy and 100 cGy conditions. In female mice at 7 months of age, there was a trend towards increased freezing after 100 cGy irradiation (p = .0561) (Fig. 1*A*). Radiation did not have a significant effect on freezing relative to a novel environment or a cued tone response in either sex (Fig. 1*B*). The second cognitive test used was a novel object recognition paradigm, which depends on multiple areas of the brain. One-way ANOVA revealed a significant change in the males [F (2,34) = 11.99, p<.0001] and post-hoc showed a decrease in exploratory time spent with the novel object for both the 10 cGy and 100 cGy irradiated male groups (Fig. 1*C*). A Student’s t-test showed significant loss of novel object recognition in the female group exposed to 100 cGy (p<.0001).

**Figure 2:**
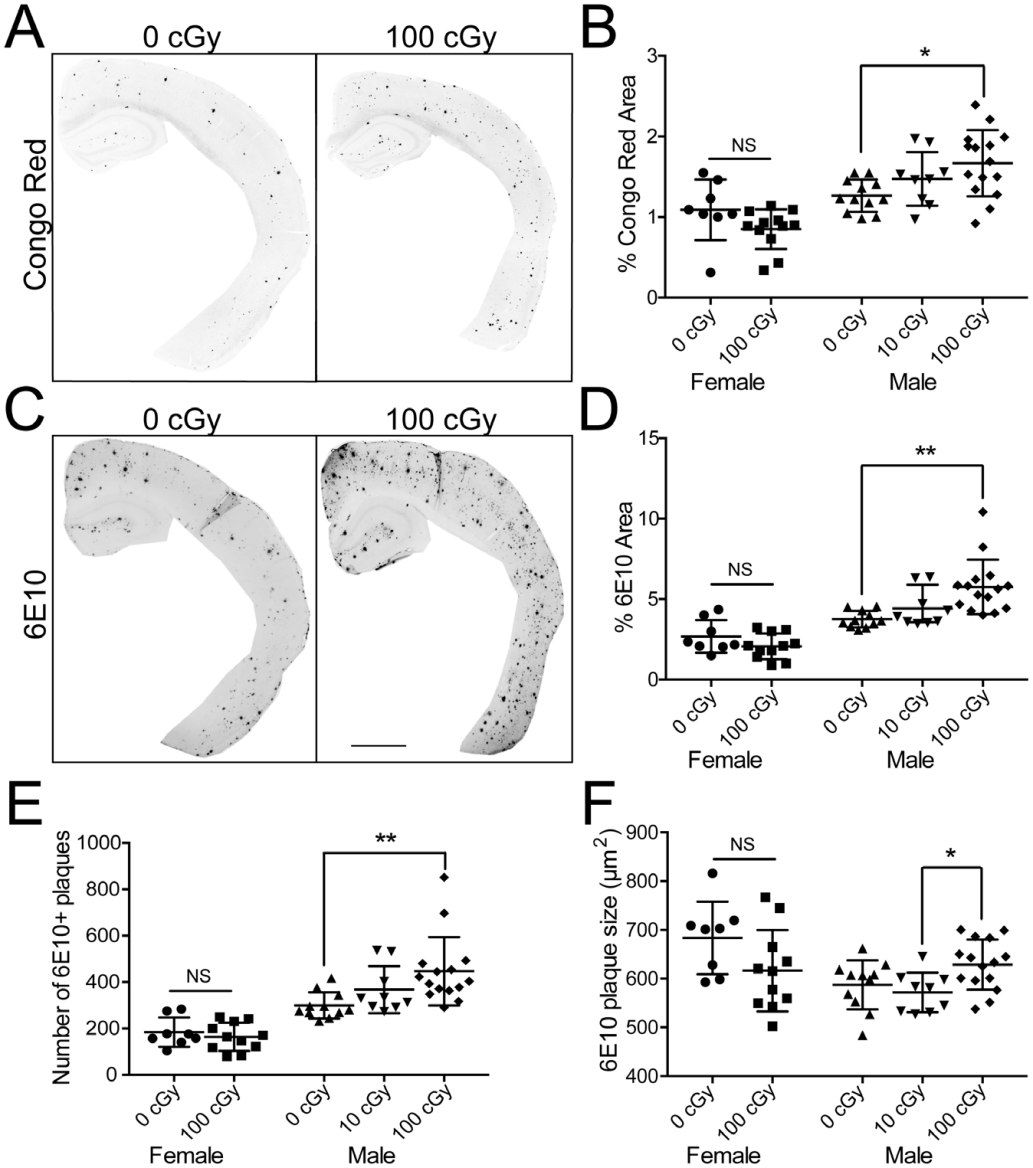
Immunohistochemical staining for Congo red and 6E10 increases after ^56^Fe particle irradiation. (*A, C*) Representative images of half male brains stained for Congo red (*A*) or 6E10 (*C*) 6 months after 0 cGy or 100 cGy ^56^Fe particle radiation. Scale bar is 1 mm. (*B, D*) Quantitative measurement of percent plaque area assessed with Congo red (*B*) and 6E10 (*D*). In addition, total number of individual 6E10 positive plaques (*E*) and the average size of plaques (µm^2^) (*F*) was determined. Each dot represents a single animal measured as percent area of the cortex and hippocampus combined. Data was analyzed with Student’s t-test for the females and one-way ANOVA with a Bonferroni post test for the males. Data displayed as mean ± SD, *n* = 8–14 animals per dose. *P<.05, **P<.01.

The radiation induced defects in learning and memory prompted us to examine if there were any alterations of Aβ pathology. Figure 2 shows results from two different kinds of amyloid stains. Congo red was used to stain dense fibrillar plaques (Fig. 2*A, B*) and 6E10, which recognizes an epitope within amino acid residues 1–16 of Aβ, labels fibrillar and non-fibrillar Aβ (Fig. 2*C, D*). At 9.5 mo of age, exposure of male mice to 100 cGy of radiation was sufficient to cause a significant increase of 38.0% in Congo red- [F(2,33) = 4.839, p = .014] (Fig. 2*B*) and a 53.8% increase in 6E10- [F(2,32) = 8.132, p = .0014) (Fig. 2*D*) labeled plaque burden (percent area). The 7 mo-old females did not show any significant difference in Congo red (p = .1011) or 6E10 (p = .1585). Using 6E10 labeling, male mice exposed to ^56^Fe particle radiation also showed a significant increase of 300 ± 56 to 447 ± 147 (mean ± SD, p = .0044) (Fig. 2*E*) in the average number of plaques after 100 cGy irradiation. Additionally, there was a trend towards larger plaque size (587 ± 50 to 628 ± 51 µm^2^, mean ± SD, p = .052) (Fig. 2*F*) in the males irradiated with 100 cGy compared to controls (0 cGy). Females did not show any changes in plaque size or number with radiation.

**Figure 3:**
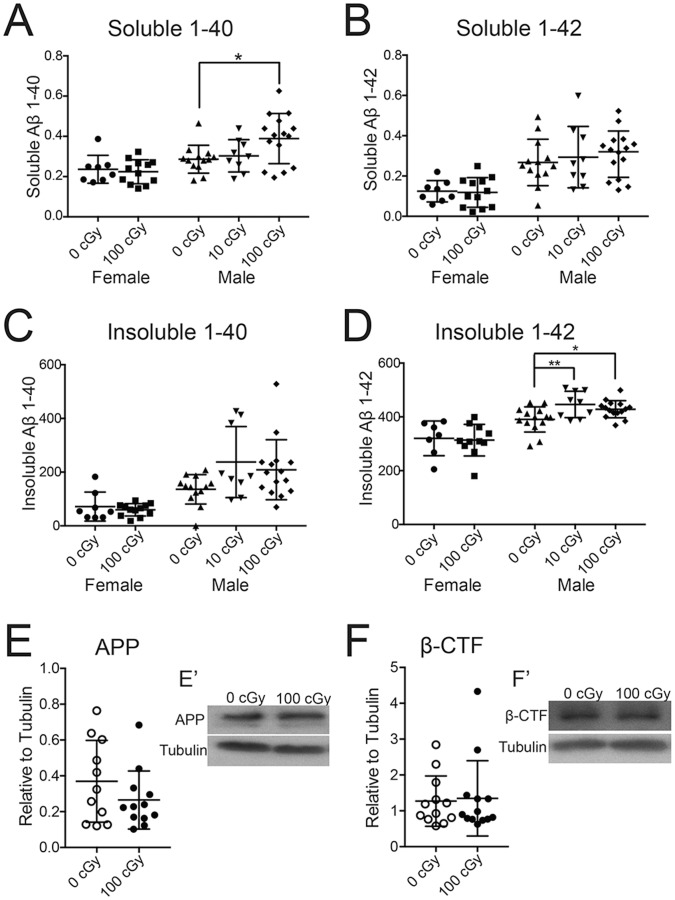
Radiation increases select Aβ isoforms but has no effect on APP processing. Dot plot analysis of soluble Aβ40 (*A*), Aβ42 (*B*) and insoluble Aβ40 (*C*) and Aβ42 (*D*). Each dot represents one animal. Data was analyzed with Student’s t-test for the females and one-way ANOVA with a Bonferroni post test for the males. (*E, F*) Male 0 cGy and 100 cGy APP (*E*) and β-C terminal fragment (*F*) protein levels were measured via Western blot and standardized to α-tubulin. Representative images of blots are present in *E’* and *F’*. Results were analyzed with Student’s t-test. Data displayed as mean ± SD, *n* = 8–14 animals per dose. *P<.05, **P<.01.

To strengthen our histology data and determine whether different forms of Aβ were altered after radiation, we prepared soluble and insoluble fractions of homogenized hemibrains and used ELISAs specific for Aβ peptides with C-terminals of 40 or 42 (Fig. 3). For the soluble fraction, there was a significant 35.9% increase in Aβ40 levels with 100 cGy radiation in male mice compared to non-irradiated controls by one-way ANOVA [F(2,34) = 4.332 p = .0211] (Fig. 3*A*). Moreover, male mice showed significant 14.8% and 10.2% increases in concentrations of Aβ42 in the insoluble fraction at both 10 and 100 cGy, respectively [F(2,36) = 6.253 p = .0047] (Fig. 3*D*), and a trend (p = .09) toward increased levels of insoluble Aβ40 after irradiation (Fig. 3*C*). No statistically significant effects were observed for Aβ40 or Aβ42 concentrations in samples prepared from female mice.

The increases found in the insoluble fraction (Fig. 3*D*) confirm our IHC results of Aβ accumulation in the males (Fig. 2). The increase in different Aβ isoforms suggests possible changes in the production of the amyloid precursor protein (APP) or increased cleavage of APP as measured by the β-secretase cleavage product (β-CTF). To determine if radiation influenced either of these processes, we measured levels of APP and β-CTF by Western blot in male mice exposed to 100 cGy ^56^Fe particles. As shown in Figures 3*E and 3F*, no changes in levels of these two species were observed relative to unirradiated controls. This suggests that the observed increases in Aβ were not due to increased APP production or processing of amyloid.

**Figure 4:**
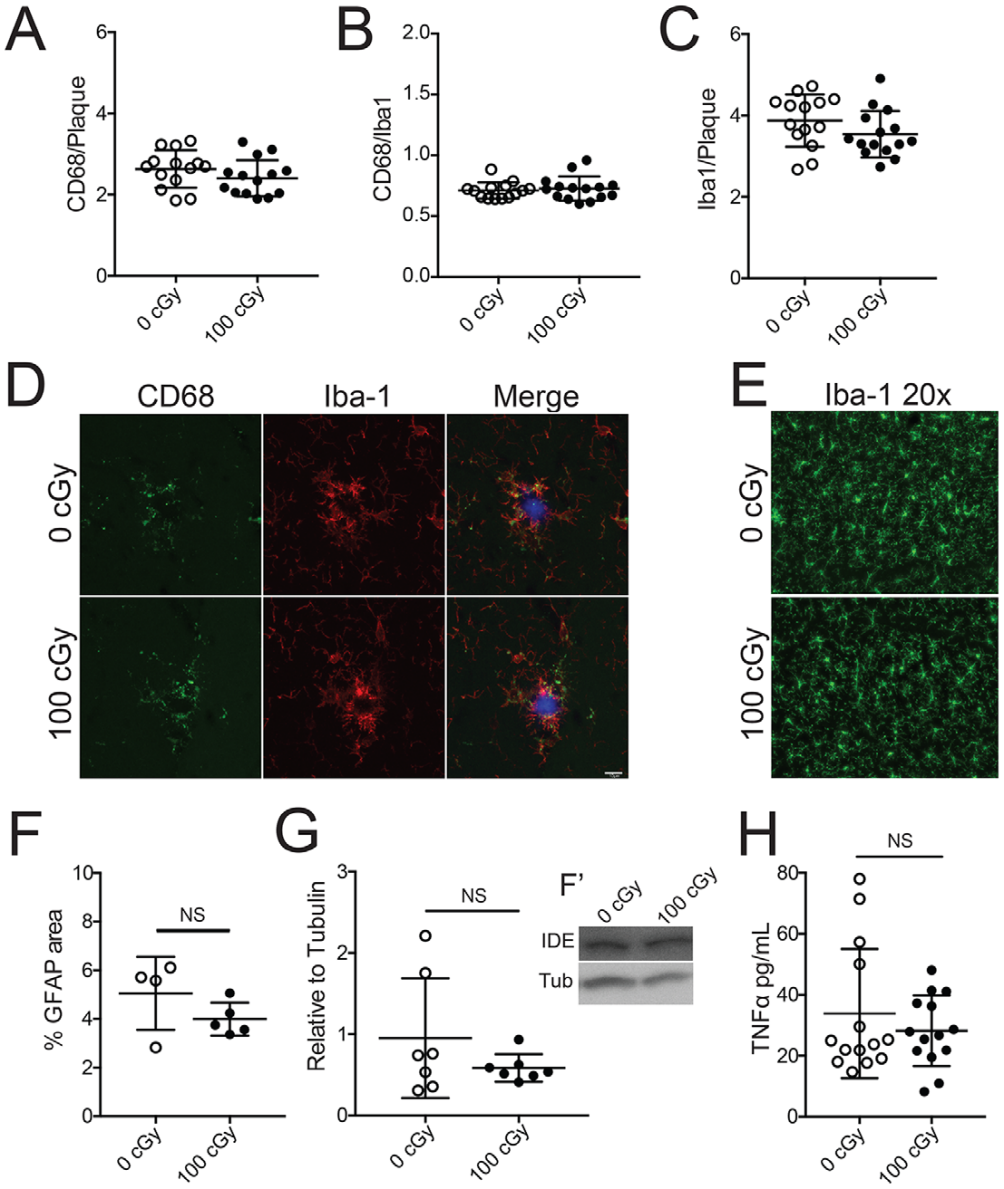
There is no change in glial activation after ^56^Fe particle irradiation. (*A*) CD68 area was normalized to individual plaque area to account for differences in plaque size. 12 plaques in each mouse were analyzed and averaged together to compare male control and 100 cGy irradiated mice. (*B*) CD68 was also normalized to the total Iba-1 microglia area around the plaque to account for potential changes in microglia number. (*C*) Iba-1 area was standardized to plaque area. Each dot represents a single animal. (*D*) Visual representation of CD68/Iba-1 staining around a plaque. Images acquired at 40x magnification, scale bar is 5 µm. (*E*) Representative hippocampal images taken to demonstrate Iba-1+ microglial morphology. Images acquired at 20x magnification, scale bar is 10 µm. (*F*) Astrocyte activation was measured using GFAP percent area measurements in the cortex (*n* = 4–5 mice per dose). (*G*) Insulin Degrading Enzyme (IDE) protein level was measured and quantified via Western blot analysis. IDE levels were normalized against α-tubulin as a loading control (*n* = 7 mice per dose). Representative images are shown in *G’*. (*H*) Protein levels of TNFα were quantified via ELISA. Data is presented as mean ± SD. The results were analysed with Student’s t test, *n* = 13–14 mice per dose in *A, B, C* and *H*.

The increase in Aβ observed by IHC and ELISA, but lack of evidence for alteration of amyloid processing, directed us to investigate other mechanisms. Due to lack of change in the female mice we elected to focus on samples from males irradiated at 100 cGy for these analyses. Microglia are principle players in CNS inflammation, which has been proposed to be an important driver of amyloid deposition. In addition, they are implicated in phagocytosis and control of Aβ [28]. We sought to identify if there was a change in the association of microglia with plaques or alterations in their level of activation that might relate to increased plaque accumulation following radiation (Fig. 4). CD68 is a commonly used marker that is upregulated in activated microglia [29] and is indicative of a phagocytic state. We did not observe any increase in CD68 area, normalized to plaque area or total Iba-1+ area, after 100 cGy radiation (Fig. 4*A, B*). Similarly, there was no effect of radiation on total Iba-1+ microglia area associated with plaques (Fig. 4*C*). Figure 4*D* contains representative images of CD68+/Iba-1+ microglia around plaques. General microglial morphology based on Iba-1 staining appeared similar in control and irradiated brain (Fig. 4*E*). Moreover, there was no significant change (p = .19) in cortical area covered by GFAP (Fig. 4*F*). To measure the ability of microglia to degrade Aβ, we quantified one of the key enzymes associated in that process, insulin degrading enzyme (IDE) [30] (Fig. 4*G*). There was no statistical difference between the control and irradiated mice when analyzed with a Student’s t-test (p = .22). Lastly, we investigated the amount of the inflammatory cytokine TNFα (Fig. 4*H*). We did not detect any difference between irradiated and control levels (p = .39). Taken together, these results demonstrate no clear evidence of increased glial activation 6 months after 100 cGy radiation exposure.

**Figure 5:**
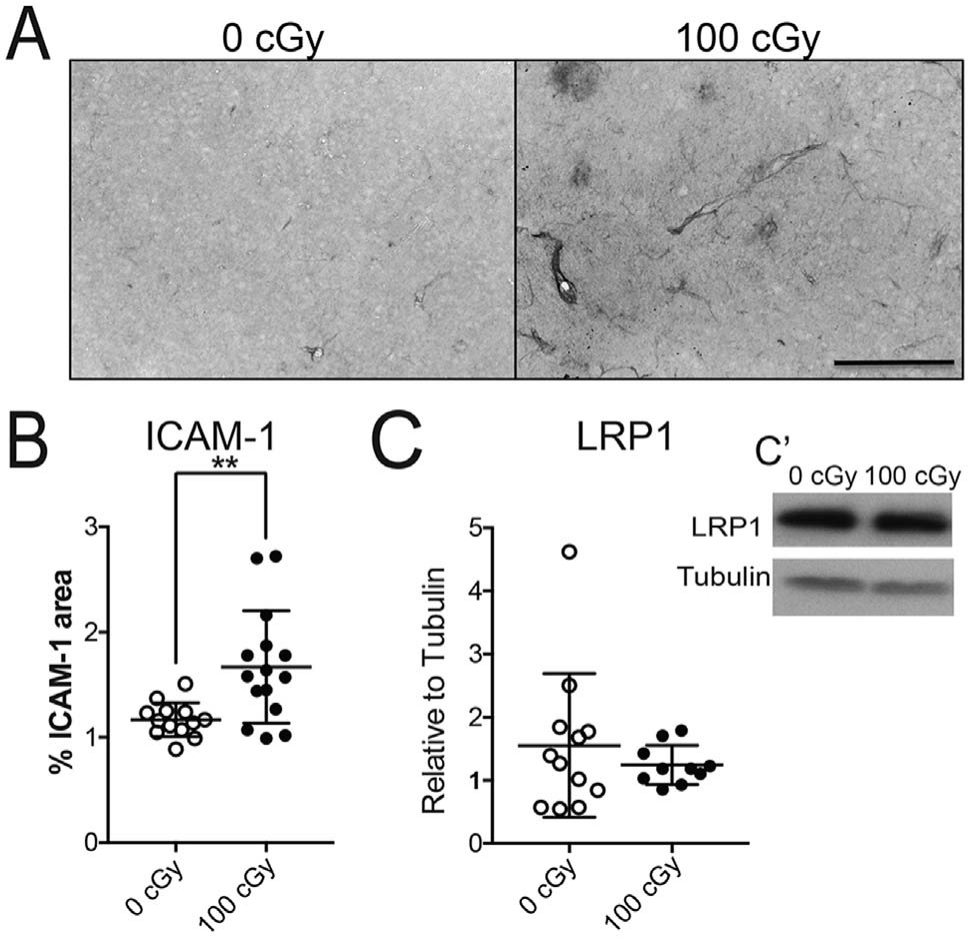
^56^Fe particle radiation causes endothelial activation. (*A*) Representative images of ICAM-1 staining. Pictures are at 20x magnification and the scale bar is 10 µm. (*B*) ICAM-1 area was measured as percent total area in the entire cortex in 2 serial sections with the results being averaged together. Each dot represents a single animal. (*C*) Protein samples were analyzed for LRP1 using Western blot. LRP1 levels were standarized against α-tubulin as a loading control. Representative immunoblot image is present in *C’*. Data is presented as mean ± SD. Results were analysed with a Student’s t-test. *n* = 13–14 animals per dose.

Due to the importance of Aβ clearance out of the brain through the BBB [20] we next examined vascular alterations in the irradiated animals. Sections were stained with ICAM-1, a marker of endothelial activation (Fig. 5). ICAM-1 is also thought to be an indirect marker of CNS damage or inflammation that we have previously demonstrated in irradiated mouse CNS [4], [31]. Relative to control tissue, a significant increase in ICAM-1 total staining through the cortex was observed after 100 cGy radiation as judged by Student’s t-test (p = .0031) (Fig. 5*A, B*). To begin to assess transport of Aβ out of the brain, levels of low-density lipoprotein receptor-related protein 1 (LRP1) were quantified in tissue samples by Western blot. LRP1 is a critical protein involved in binding Aβ and trafficking it out of the brain [32] that can be modulated by peripheral inflammatory signals [33]. Even though radiation resulted in increased endothelial activation, we did not observe any difference in LRP1 protein level 6 months after 100 cGy ^56^Fe particle irradiation (Fig. 5*C* *)*.

## Discussion

Here we report that GCR caused enhanced AD plaque pathology. To our knowledge, this is the first report of radiation being associated with enhanced plaque pathology in an AD mouse model. In addition to disease acceleration, we observed that low HZE doses are able to cause cognitive impairment as measured by contextual fear conditioning and novel object recognition in APP/PS1 tg mice (Fig. 1). While contextual fear conditioning and, to a certain extent, novel object recognition are dependent on an intact hippocampus, the cued tone freezing response is thought to measure hippocampal independent memory [34], [35]. The lack of impairment in tone mediated freezing demonstrates that the cognitive dysfunction we observe can be, at least in part, traced to hippocampal mediated memory processes. This is consistent with other reports on the effect of radiation impacting hippocampal dependent memory [7], [36]. Because we did not run parallel studies with wild-type control mice, we do not know whether cognitive impairment resulted from radiation alone or represented a synergy between radiation and mutant AD gene expression in these mice. HZE irradiation alone can lead to cognitive deficits in wild-type mice [7]; however, the only report of deficits in contextual fear conditioning or novel object recognition with C57BL/6 mice required 200 or 300 cGy iron [37]. Unfortunately, differences in mouse strain, timing, and radiation beam energy limit our ability to extrapolate from these studies. Multiple possible radiation induced effects might contribute to cognitive dysfunction in our model. One example is a defect in neurogenesis, which has been documented in response to traditional radiotherapy [38] as well as exposure to ^56^Fe particles [5], [7], [39]. In addition to neuronal proliferation defects, impaired cognition could also result from inhibition of long-term potentiation (LTP) [40], an effect which has been reported with ^56^Fe particle irradiation in the APP23 transgenic mouse model of AD [41].

In addition to behavioral deficits, we saw enhanced Aβ plaque accumulation as judged by two different markers. 6E10 showed an increase in total deposited Aβ levels and Congo red showed an increase in aggregation of plaques into dense fibrils. These results were further confirmed by ELISA data (Fig. 3). Aβ plaque staining is used to gauge progression and stage AD pathology [12]. The increases observed in soluble Aβ and insoluble plaque deposition suggest that GCR caused more rapid progression of AD, at least for male mice. The female group was sacrificed at an earlier age than the male mice due to concerns related to several female mice dying early. Given the small number that died, we do not know whether this was related to radiation; our goal was to have a large enough cohort for behavioral and tissue analysis. Thus the male and female groups are not comparable. Moreover, APP/PS1 female mice are known to have different plaque dynamics then males [42]; therefore it is not possible to draw specific conclusions on gender difference of ^56^Fe particle radiation.

The doses used in this study are comparable to those astronauts will see on a mission to Mars [2], [3], raising concerns about a heightened chance of debilitating dementia occurring long after the mission is over. Increased plaque progression could be due to a variety of mechanisms. A primary mechanism of radiation injury is DNA damage and reactive oxygen species production [38], [43] that can contribute to overall cell dysfunction. In addition, radiation is also known to cause glial activation and inflammatory cytokine production [4], both of which have been implicated in neurodegenerative diseases like AD [44]. In our study, GCR exposure could amplify the chronic inflammatory AD state and speed up pathology. However, we did not find clear evidence of neuroinflammation using markers previously shown to be elevated using higher doses of gamma and HZE irradiation [4], [5], [45]. However, subtle inflammatory changes could be occurring that we were not able to visualize by conventional immunohistochemical methods. Additionally, investigators have shown there is a biphasic pattern of inflammatory cytokines over several months after irradiation [4], [45], suggesting the possibility that significant changes at another time point might have been missed. Indeed, Encinas et al. observed accumulation of Iba1+ microglia in the hippocampal subgranular zone 6 h post 100 cGy ^56^Fe radiation exposure. This effect was not seen 24 h or 3 weeks after irradiation [46]. This observation is consistent with microglial reaction to hippocampal neural precursor cells undergoing apoptosis in response to radiation [47], and suggests that neuroinflammation might occur in our model at an acute time point.

Microglia have been implicated in plaque maintenance in a number of models [28], [44], [48], [49]. Although radiation induced changes in microglia might result in increased plaque deposition, we did not find alteration in several measures related to microglial function. Moreover, we observed no increase in the Aβ degrading enzyme IDE as pathology worsens after 100 cGy irradiation (Fig. 4*F*). IDE is an enzyme that is present in several CNS cell types [30]. Importantly, it is thought that microglia can secrete it to degrade extracellular Aβ [50]. One could argue that the lack of increased IDE is a significant finding since it would be expected that as pathology worsens, there should be an upregulated response. It is important to note that IDE is not the only protease implicated in Aβ degradation. Other proteases like neprilysin or MMP9 could potentially be involved [30].

An additional hypothesis is that radiation causes vascular defects, which impair proper clearance of Aβ. Clearance through the vasculature has been shown to be crucial [20] and alterations by various means can result in increased pathology [33]. Radiation led to increased ICAM-1 staining and vascular dysfunction, including increased permeability [4], [31], [51]. We found significant increases in ICAM-1 staining in male mice 6 months after exposure to 100 cGy ^56^Fe particles (Fig. 5). It is tempting to speculate that radiation-induced vascular changes alter the transport of Aβ out of the brain. Even though we did not observe any change in LRP1, which is associated with Aβ removal from the brain and known to be influenced by inflammatory stimuli [33], there are additional transporters found at the BBB that might have a role in Aβ removal [20]. Ultimately, Aβ tracer studies will be required to definitively demonstrate impaired clearance in irradiated mice.

In conclusion we have demonstrated that 100 cGy of ^56^Fe particle radiation can cause cognitive impairment as well as increased Aβ plaque pathology in APP/PS1 mice, without clear changes in glial activation. Additionally, the elevation of ICAM-1 expression in irradiated mice raises the possibility that vascular changes might underlie radiation-induced amyloid accumulation. These pathological increases are particularly concerning for astronauts who will be exposed to GCR in upcoming deep space missions. In this regard, one major caveat of our model is that mice were subjected to acute exposures with a single HZE species. It is not known how the CNS will respond to the complex and chronic low-dose GCR environment of space. Moreover, astronauts will not likely be familial AD carriers. Therefore, while many of the pathological processes are believed to be similar, this model does not reflect the complete human condition. However, for the one aspect we can replicate, the accumulation of Aβ, our findings demonstrate that whole body exposure to ^56^Fe particle HZE radiation enhances pathological processes associated with progression of AD.
